# Frontolateral transfalcine approach to olfactory groove meningioma: rationale for surgical selection. Two-dimensional video

**DOI:** 10.3171/2025.10.FOCVID25173

**Published:** 2026-01-01

**Authors:** Erik Burgos-Sosa, Vera Vigo, Yuanzhi Xu, Byron Hontiveros, Muhammad Reza Arifianto, Matei A. Banu, Juan C. Fernandez-Miranda

**Affiliations:** Department of Neurosurgery, Stanford University Medical Center, Stanford, California

**Keywords:** olfactory groove meningioma, frontolateral approach, olfactory nerve preservation

## Abstract

Olfactory groove meningiomas represent a surgical challenge due to their intimate relationship with the anterior cranial fossa, olfactory tracts, and critical neurovascular structures. In this video, the authors present a microsurgical resection of an olfactory groove meningioma through a right frontolateral approach. This technique provides a wide operative corridor with minimal frontal lobe retraction, early devascularization of the tumor, and anatomical preservation of the contralateral olfactory tract. Stepwise dissection, relevant microsurgical anatomy, and surgical pearls are demonstrated. The video highlights the safety and rationale of this approach for selected anterior skull base meningiomas with emphasis on olfactory function preservation.

The video can be found here: https://stream.cadmore.media/r10.3171/2025.10.FOCVID25173

## Transcript

### 0:21 Patient Clinical Presentation and Imaging.

This is the case of a 41-year-old female patient with an incidental finding of an olfactory groove meningioma that has grown in follow-up. The patient has intact olfaction. We can see here the imaging findings showing a medium-sized olfactory groove meningioma with no significant brain swelling.

### 0:45 Surgical Approach Options.

We discussed all the potential alternatives, the approaches that can be used for this particular lesion. We’ll include the endoscopic endonasal approach, which we do not favor in this case because of the large skull base defect that we’ll create and the sacrifice of the sense of smell.^1–3^

We can discuss the transnasal approach, which would also likely sacrifice the sense of smell^3^ and is a more invasive approach than the alternative: pterional, orbitozygomatic, or frontolateral approach.

In this particular case, we’re going to employ what we call a frontolateral approach, which can be done through an eyebrow incision,^4^ or through a scalp incision behind the hairline, which is what we chose in this particular case.

This frontolateral or supraorbital approach provides direct access to the anterior skull base and allows the transsection of the falx to access the contralateral side, aiming to preserve the sense of smell in this particular case by preserving the contralateral olfactory tract. And that’s why we chose this particular approach in this case.

### 1:53 Anatomical Essentials.

We can discuss some of the key anatomy: opticocarotid cistern, olfactory tract, olfactory sulcus, the area of the cribriform plate, the falx, and the relationship of the falx with the olfactory bulb.^5^

### 2:07 Description of the Surgical Setup.

We describe a setup with a basic supine position with head rotated approximately 30° to the contralateral side and with a lot of extension to facilitate gravity retraction of the frontal lobes.

### 2:21 Surgical Procedure.

Simple craniotomy, avoiding the frontal sinus. We did not remove the roof of the orbit, but it’s a medially extended frontal craniotomy with a subfrontal approach. Intradurally, we access the cisterns; the opticocarotid cistern is accessed early on. This greatly relaxes the frontal lobe, which allowed doing this operation without the need for any fixed retraction. The brain is nicely protected with cottonoids. We can start seeing the implantation of the tumor into the anterior skull base, and we of course start devascularizing the tumor at its base, and we can see here we can very easily access the falx, coagulate any potential falcine branches coming from the area. We can see the right olfactory tract; it is very stretched by the tumor. We do not intend to preserve this olfactory tract, so we sacrifice it upfront. This allowed us to get excellent access to the tumor. We can see the right olfactory bulb. Again, we do not try to preserve the olfaction in this side, but our aim is to preserve the contralateral olfactory nerve. By coagulating this olfactory bulb, we can access the ipsilateral olfactory sulcus and make sure that it is completely clean of tumor. We continue devascularizing the tumor and then just performing the standard intracapsular debulking.

Again, we avoid fixed retraction with good fluid drainage on the cisterns and head positioning; we can relax the brain nicely. Very careful with the orbitofrontal arteries. Don’t be tempted to coagulate these branches. These are still important to provide vascular supply to the fronto-orbital region and they are not tumor branches, although they can provide vascular supply; they can be selectively preserved. We are now coagulating and transsecting the falx, and this allows us to access the contralateral aspect of the tumor carefully, not to injure the contralateral olfactory bulb.

We can now identify the falcine artery. This is now being coagulated. This is a major source of blood supply in this case. And after careful debulking, we can start identifying the contralateral olfactory nerve. We have direct access to the contralateral side. We preserve not just the olfactory nerve, but the microvascular supply to this nerve. We are now dissecting crista galli and the dura that surrounds it, and the falx implantation. This allows me to get better access. I respect the arachnoid planes because, again, I want to preserve the arachnoid that covers the olfactory nerve. I placed a bit of Gelfoam with nicardipine to prevent vasospasm on the microvasculature of the left olfactory nerve. Again, a major goal of the operation is to preserve the olfaction on the other side. And now the tumor is being detached from the contralateral arachnoid of the fronto-orbital region. We see another fronto-orbital branch that we are meticulously dissecting and preserving. Using the arachnoid planes, we can dissect the proximal part now of the olfactory tract. Again, we have to be careful in this area, preserve arachnoid planes, preserve the microvasculature. Another piece of Gelfoam with nicardipine to treat that vasculature and prevent vasospasm of the microvessels to the olfactory nerve. Last bits of tumor are stuck to that fronto-orbital branch. We use, again, arachnoid dissection planes to fully resect the last bits of tumor that are extending toward the contralateral olfactory sulcus. We coagulate the implantation of the tumor, careful not to coagulate the contralateral olfactory sulcus. The dura on the anterior skull base all the way to the cribriform plate on the patient’s right side is being transected. We also drill the skull base without entering to the sinuses.

### 6:35 Postoperative Course.

The patient had no complications. Fortunately, the sense of smell was very well preserved. The patient was discharged on postoperative day 2.

### 6:45 Conclusions.

This case highlights how a traditional frontolateral approach can be the best choice for this medium-sized olfactory groove meningioma, where we want to preserve the sense of smell. Thank you.

## Disclosures

Dr. Fernandez-Miranda reported personal fees from Medtronic, Hotry, and KLS Martin, and nonfinancial support from Stryker and Storz outside the submitted work.

## Author Contributions

Primary surgeon: Fernandez-Miranda. Assistant surgeon: Banu. Editing and drafting the video and abstract: Fernandez-Miranda, Burgos-Sosa, Xu, Hontiveros, Reza Arifianto. Critically revising the work: Fernandez-Miranda, Burgos-Sosa, Vigo, Xu, Banu. Reviewed submitted version of the work: Fernandez-Miranda, Burgos-Sosa, Vigo, Xu, Banu. Approved the final version of the work on behalf of all authors: Fernandez-Miranda. Supervision: Fernandez-Miranda, Xu.
